# A New Perspective to Longstanding Challenges with Outpatient Hyperkalemia: A Narrative Review

**DOI:** 10.1177/20543581221149710

**Published:** 2023-01-12

**Authors:** Michael Chiu, Amit X. Garg, Louise Moist, Arsh K. Jain

**Affiliations:** 1 Division of Nephrology, Department of Medicine, London Health Sciences Centre, Western University, London, ON, Canada; 2 Kidney Clinical Research Unit, London Health Sciences Centre, London, ON, Canada; 3 Department of Clinical Epidemiology and Biostatistics, Western University, London, ON, Canada

**Keywords:** hyperkalemia, standard of care, chronic kidney disease, laboratory reporting, quality improvement

## Abstract

**Purpose of Review:**

Outpatient hyperkalemia is a common problem with potentially deadly consequences. Potassium level thresholds to treat outpatient hyperkalemia are unstandardized and variable, leaving health care providers to rely on their own clinical judgment. This narrative review highlights the challenges of outpatient hyperkalemia management and includes recommendations for future studies that may standardize treatment, improve patient outcomes, and optimize health care utilization.

**Sources of Information:**

PubMed, Google Scholar, and the reference lists of identified articles were used to include English, peer-reviewed studies and guidelines for this review.

**Methods:**

This narrative review examines outpatient hyperkalemia from both a laboratory and clinical perspective. In addition to peer-reviewed literature, guidelines and expert consensus statements were included to highlight the inconsistencies and paucity of evidence that health care providers rely on to make clinical decisions.

**Key Findings:**

There are multiple reasons why outpatient hyperkalemia management is both challenging and sub-optimal. Clinicians must discern if the potassium level result is accurate and, if so, does the result warrant referral to the emergency department. Factitious hyperkalemia, or falsely elevated potassium level results due to analytical errors, occurs frequently, but there are no ways to identify it other than for hemolyzed samples. Additionally, guidelines and expert panels are inconsistent on the thresholds for treatment and the management of hyperkalemia. Finally, there are inconsistencies between laboratories as to when and how providers are notified of results, and the suggested thresholds for urgent management. A study that integrates the expertise of clinical biochemists and clinicians is needed to inform evidence-based guidelines for the management of outpatient hyperkalemia.

**Limitations:**

This was a comprehensive review of what is known and what still needs to be understood for the management of outpatient hyperkalemia. A formal tool to assess the quality of the included studies was not used and selection bias may have occurred.

## Abrege

**Raison de la revue:**

L’hyperkaliémie ambulatoire est fréquente et peut avoir des conséquences mortelles. Les seuils de potassium justifiant le traitement de l’hyperkaliémie ambulatoire varient et ne sont pas normalisés; les professionnels de la santé doivent par conséquent s’en remettre à leur propre jugement clinique. Cette revue narrative met en évidence les défis liés à la prise en charge de l’hyperkaliémie ambulatoire et comprend des recommandations pour de futures études pour standardiser le traitement, améliorer les résultats des patients et optimiser l’utilisation des soins de santé.

**Sources d’information:**

PubMed, Google Scholar et les listes de références des articles répertoriés ont été utilisés pour inclure les études évaluées par les pairs et les lignes directrices rédigées en anglais.

**Méthodologie:**

Cette revue narrative examine la prise en charge de l’hyperkaliémie ambulatoire du point de vue clinique et paraclinique. En plus des articles évalués par les pairs, on a inclus des lignes directrices et des déclarations de consensus d’experts afin de mettre en évidence les incohérences et la rareté des données probantes sur lesquelles s’appuient les prestataires de soins de santé pour prendre des décisions cliniques.

**Principaux resultants:**

Plusieurs raisons peuvent expliquer pourquoi la prise en charge de l’hyperkaliémie ambulatoire est à la fois difficile et sous-optimale. Les cliniciens doivent juger eux-mêmes si le résultat du potassium est exact et, dans l’affirmative, déterminer s’il justifie l’aiguillage du patient vers les urgences. L’hyperkaliémie factice, soit des taux de potassium faussement élevés en raison d’erreurs analytiques, est fréquente, mais il n’existe aucun moyen de l’établir à l’exception des échantillons hémolysés. Aussi, les lignes directrices et les groupes d’experts ne s’entendent pas sur les seuils de traitement et de prise en charge de l’hyperkaliémie. Enfin, on observe des incohérences entre les laboratoires quant au moment et à la façon dont les prestataires sont informés des résultats, de même qu’en ce qui concerne les seuils suggérés pour une prise en charge urgente. Une étude intégrant l’expertise des biochimistes cliniques et des cliniciens est nécessaire pour éclairer des lignes directrices fondées sur des données probantes pour la prise en charge de l’hyperkaliémie ambulatoire.

**Limites:**

Il s’agit d’un examen complet de ce que l’on connaît et de ce qui doit encore être compris au sujet de la prise en charge de l’hyperkaliémie ambulatoire. Des biais de sélection pourraient s’être introduits puisqu’aucun outil formel n’a été utilisé pour évaluer les études incluses.

## Why is this review important

Outpatient hyperkalemia is a common manifestation of medications and medical conditions such as chronic kidney disease. Unfortunately, guidelines on how health care providers are informed about hyperkalemia in the outpatient setting and how they should act upon the result are inconsistent. Therefore, there are variable practice patterns, patient outcomes, and health care utilization. This review summarizes the gaps in current knowledge in acute and severe outpatient hyperkalemia and serves as a call to action on the research needs that will inform best practice to develop management and reporting guidelines. Chronic, low-grade hyperkalemia management is not within the scope of this review.

## What are the key messages

The gaps in outpatient hyperkalemia include inability to identify factitious hyperkalemia, the unknown threshold for life-threatening hyperkalemia that requires urgent emergency department (ED) management, and the inconsistent laboratory reporting process for hyperkalemia.

## Clinical Case

John Smith is a 60-year-old man followed for diabetic kidney disease. His medications include perindopril 4 mg daily, dapagliflozin 10 mg daily, sitagliptin-metformin 50-500 mg BID, and amlodipine 5 mg daily. His creatinine is 148 µmol/L (eGFR: 48 mL/min/1.73 m^2^) and potassium level is 4.6 mmol/L. Urinalysis reveals no blood with a urinary albumin-to-creatinine ratio (ACR) of 117 mg/mmol. The perindopril is increased to 8 mg daily and the repeat creatinine 1 week later is 124 mg/mmol and the potassium level is 4.8 mmol/L. The author decided to reassess in 6 months.

At 3-month follow-up with the family doctor, the potassium level is 5.7 mmol/L. The patient is given dietary advice to restrict potassium level intake, and no change was made to the perindopril. The repeat potassium level done 1 week later is 6.1 mmol/L. The patient is started on kayexalate, and the family physician contacts the author to manage potassium level.

Mr. Smith is seen in the hospital clinic, and the repeat potassium level from the hospital lab is 3.9 mmol/L. Kayexalate is stopped. The repeat potassium level from an outpatient lab is again high at 6.0 mmol/L. Rather than making any changes, the patient is instructed to return to the hospital lab and the repeat potassium level is 4.3 mmol/L.

## Introduction

Hyperkalemia is a common clinical scenario with potentially deadly consequences. It is managed by multiple medical specialties, especially nephrology. There are recommendations for managing hyperkalemia in a hospital setting, but outpatient hyperkalemia management is more variable. Basic questions on how to treat outpatient hyperkalemia remain unknown:

- How does the provider ensure the potassium level is accurate?
- What constitutes severe life-threatening hyperkalemia that results in poor patient outcomes?
- When should these patients be sent to the ED versus treated as an outpatient?
- When and how should health care providers be alerted to hyperkalemic results?

Outpatient hyperkalemia occurs frequently and is a burden for the health care system and patients. The prevalence of hyperkalemia in the ED can be up to 8%.^1,2^ Additionally, an American study demonstrated outpatient hyperkalemia compared with normal values is associated with an over US$19,000 increase in health care costs per patient per year (due to ED visits, hospitalizations, and outpatient visits).^3^ These higher health care costs are seen globally (including North America, Europe, and Asia).^4-7^ Furthermore, hyperkalemia is a psychological burden to patients. The National Kidney Foundation conducted a survey of 488 patients with chronic kidney disease (CKD) in the United States. In the report, 38% of respondents were sent to the ED to manage hyperkalemia, 44% were “very concerned” about their potassium levels, and 59% saw hyperkalemia as an important personal issue to them.^8^

Despite the frequency and burden of outpatient hyperkalemia, clinicians do not have a standard approach to managing these patients. To our knowledge, there has not been interdisciplinary research to integrate the analytical and clinical dimensions of outpatient potassium management. This article will highlight the challenges with hyperkalemia surveillance and detection, the inconsistent guidelines on how to best treat outpatient hyperkalemia, and the unstandardized approach labs use to report hyperkalemia to providers. Given these discrepancies, a research program that includes both clinical biochemists and clinicians is necessary to ensure optimal patient outcomes and health care utilization.

## Methods

A comprehensive narrative review was performed to explore several perspectives of outpatient hyperkalemia, including laboratory analysis, clinical outcomes, and laboratory reporting. Relevant literature was searched on PubMed and Google Scholar using “hyperkalemia,” “chronic kidney disease,” and “outpatient management.” Review of the referenced literature in the included studies was also completed. The evidence behind the included guidelines and consensus statements was summarized by the authors. Only peer-reviewed and English articles were included.

## Factitious Hyperkalemia: How Do Health Care Providers Ensure They Are Treating Accurate Results?

There are several steps needed to obtain a potassium result from a blood sample, from phlebotomy to processing, analysis, and reporting. Factitious hyperkalemia, or an artificially high potassium result due to a release of intracellular potassium during the testing process, may result from errors or delays in any of these steps. This includes collection technique,^9^ fist clenching during collection,^10^ the sample on ice too long prior to processing,^11^ and delayed centrifugation^12^ (see Table 1). Unfortunately, these may occur in up to 33% of samples.^13^ Sometimes, factitious hyperkalemia is detected as a hemolyzed sample, which occurs with mechanical trauma to red blood cells to release intracellular potassium, and these results are flagged to inform providers to interpret the result with caution. The hemolyzed result can be corrected based on the hemolytic index (a spectrophotometric assessment of serum to detect hemoglobin that is also released); however, labs are unable to provide an accurate corrected result.^13^ Factitious hyperkalemia may be due to leeching of potassium out of red blood cells rather than hemolysis. There are no alerts for these scenarios, which poses an additional challenge for providers.

**Table 1.** Causes of Factitious Hyperkalemia.

Cause of factitious hyperkalemia	Mechanism
Collection technique	Increased turbulence through a small diameter needle or with negative pressure can result in mechanical trauma to red blood cells.
Fist clenching during collection	May cause release of potassium in forearm muscles or increase blood flow (resulting in turbulent mechanical damage).
Keeping sample on ice too long	The sodium-potassium ATPase is inhibited in cold temperatures. The sodium-potassium ATPase is therefore unable maintain the gradient and potassium leaks from cells.
Delayed centrifugation	As glucose is consumed, there is a reduction in available glucose that generates ATP, resulting in leakage of intracellular potassium.

Health care providers must also be aware of the different methodologies of potassium testing. Potassium can be measured from either serum, plasma, or whole blood.^13^ The cellular components of blood are separated from serum and plasma by centrifugation. In serum testing, blood is allowed to clot prior to centrifugation whereas plasma samples are collected in anticoagulated tubes. Serum sampling may be more prone to factitious hyperkalemia since potassium is released during the clotting process in addition to the delay of processing. However, plasma potassium can be centrifuged and analyzed immediately and is therefore less prone to delays in testing.^14^ The reference intervals for serum and plasma potassium are similar. In Life Labs, a predominant outpatient laboratory in Ontario, the reference range for serum potassium is 3.5-5.2 mmol/L.^15^ In London Health Sciences Center, a tertiary care hospital in Southwestern Ontario, the reference range for plasma potassium is 3.5-5.0 mmol/L. Although the results correlate well, a study of 720 individuals with both a serum and plasma potassium value showed higher variability of results at higher potassium values.^14^ It is important to note that both serum and plasma samples are prone to hemolysis. Whole blood testing, using point-of-care gas analyzers, does not require centrifugation and can be tested immediately. It is recommended in situations where immediate results are needed (i.e. cardiac arrest) or in severe leukocytosis or thrombocytosis states (i.e. hematologic malignancies) as centrifugation results in lysis of leukocytes or platelets.^13^ Instruments in whole blood testing do not have the ability to detect hemolysis using spectrophotometric analyzers so hemolysis that may occur through blood collection or analysis is not detected.^16,17^ Therefore, serum or plasma testing is preferred in an inpatient or outpatient scenario. Unfortunately, the methodology of testing (serum vs. plasma) is not standardized between centers, so it is important for providers to understand these differences when an outpatient potassium result is received.

It is essential to identify factitious hyperkalemia before initiating treatment. Currently, the only way to identify non-hemolyzed factitious hyperkalemia is to repeat the sample. A repeat result in the normal range, without treatment, is indicative of factitious hyperkalemia. Analytical errors in potassium measurement are common. A study of outpatients in Singapore revealed that factitious hyperkalemia, defined as a potassium >5.5 mmol/L, occurred in 86% of repeated potassium samples (1362/1575 repeated samples were normal within 8 days).^18^ Although this study has limitations, it reveals that factitious hyperkalemia is a common scenario. The question remaining is: How can factitious hyperkalemia from nonhemolyzed samples be identified without having the patient repeat their blood work?

## Severe Hyperkalemia: When Is Urgent Really “Urgent”?

Normal potassium, ranging from 3.5 to 5.4 mmol/L, is essential for proper heart function. Hyperkalemia is commonly identified without patient symptoms yet severe hyperkalemia can result in cardiac arrhythmias and death.^19^ In a retrospective study of patients with hyperkalemia in the ED, 71% of patients with a potassium >6.5 mmol/L had an electrocardiogram (ECG) abnormality.^20^ Adverse events such as death, cardiac arrhythmias, and requirement of cardiopulmonary resuscitation (CPR) increased significantly in those with ECG changes. However, studies have also shown that the ECG changes do not necessarily correlate well with the level of potassium.^21,22^ Several studies have demonstrated that patients with hyperkalemia, at levels ranging from >5.0 to >6.0 mmol/L, have between 1.5 and 30 times higher risk of death, within 1 day to 18 months, compared to those with normal levels.^23,24^ There is no standard definition of severe hyperkalemia. In this narrative review, severe hyperkalemia is defined as hyperkalemia could result in death. In the aforementioned studies, the threshold for severe hyperkalemia that resulted in adverse events was inconsistent.

The rationale for urgent treatment of acute severe hyperkalemia in the ED is to reduce morbidity and mortality. Urgent treatment of hyperkalemia includes stabilizing the cardiac membrane to reduce the risk of cardiac arrhythmias with intravenous calcium.^25-30^ While this is routinely done, it is not supported by randomized controlled trials.^31^ Second, immediate treatment includes redistributing potassium. This can be done with intravenous insulin and salbutamol.^31,32^ Intravenous bicarbonate can also be used in the case of metabolic acidosis.^33,34^ Definitive treatment of hyperkalemia requires promoting excretion. Potassium excretion can be increased using diuretics, or in some cases it must be done extra-corporally with dialysis.^29^ All of these treatments are done with close cardiac monitoring as rapid changes in potassium levels can also precipitate arrhythmias.^35^ Novel binders, including patiromer and sodium zirconium cyclosilicate have also been shown to lower potassium.^36,37^ However, clinical trials for their urgent use in the ED are limited and there are ongoing studies investigating clinical outcomes (NCT04585542).^38^ Even when a patient presents to the ED, there are no standard guidelines for health care providers to manage hyperkalemia. In a study of hyperkalemic outpatients (median potassium of 6.3 mmol/L) presenting to ED, there were 43 different treatment permutations utilized within the first 4 hours.^21^

Outpatient management for hyperkalemia is more challenging since the treatment options used in the ED are unavailable in the outpatient setting. Strategies to correct hyperkalemia include dietary modification to reduce potassium intake^39^ and use of potassium binders.^36,37,40,41^ However, these strategies do not allow for the patient to be closely monitored to manage the risks of hyperkalemia. Therefore, many providers decide to send their patient to ED for monitored management. Unfortunately, there are no agreed-upon evidence-based approaches for outpatient hyperkalemia management, including when to send patients to the ED, and existing guidelines from expert consensus are inconsistent. The guidelines from KDIGO,^25^ the Canadian Cardiovascular Society,^26^ UK Renal Association,^27^ and European Guidelines for Resuscitation^28^ have different thresholds to send patients to ED. Expert opinions from the French Society of Cardiology^30^ and Italian Society of Nephrology are similarly discrepant.^42^ Additionally, the recommendations are based on small observational studies or studies that do not investigate outpatient hyperkalemia outcome thresholds (Table 2). Health care providers are therefore left to use their best clinical judgment on how to treat patients with severe outpatient hyperkalemia.

**Table 2.** Management Suggestions From Expert Panels.

Expert guidelines	Urgent potassium threshold to send patient to ED	Limitations of guideline evidence
Canadian Cardiovascular Society 2016	>6.0 mmol/L	Based on thresholds from EMPHASIS-HF^35^ and CHARM^36^ studies. There were few patients with hyperkalemia (<5% of trial patients) and did not study hyperkalemia outcomes.
KDIGO 2020	>6.0 mmol/L or any ECG changes	Based on convenience samples and small studies^16,37^ (n = 23 and n = 188). Studies only investigated acute management of inpatients rather than outpatient outcomes.
Renal Association 2020	>6.5 mmol/L OR Acutely unwell patients if >5.5 mmol/L, particularly in presence of acute kidney injury	Based on a small study (n = 23) that lacked power to determine outcome^37^ and an epidemiological study^38^ (n = 195 178) that demonstrated that levels >6.0 resulted in greater mortality and health care utilization, but did not provide a threshold to suggest ED referral.
European Guidelines for Resuscitation 2021	>6.5 mmol/L	Only indicates threshold for emergency treatment. Based off a small retrospective study^16^ (n = 188) that investigated adverse events of inpatients who had hyperkalemia.
Consensus statements	Urgent potassium threshold to send patient to ED	Limitations of consensus statement
French Society of Cardiology	>6.0 mmol/L	Expert opinion—did not cite a study as evidence.
Italian Society of Nephrology	>6.5 mmol/L—urgent intervention needed	Cited cohort study^39^ (n = 923) of patients admitted to hospital that investigated outcomes of hyperkalemia (K > 6.5 mmol/L).

*Note.* ED = emergency department.

## Potassium Reporting: When and How Should Hyperkalemia Be Reported to the Clinician

Since hyperkalemia is potentially deadly, health care practitioners must be alerted about high potassium levels in an expeditious manner. However, when and how health care providers should be notified toward a clinically important result is unclear. For example, the current Ontario Association of Medical Laboratories reporting guidelines grade hyperkalemia as an “Alert” value if the potassium is between 6.2 and 6.6 mmol/L. At this value, the reporting laboratory will alert the provider to the result, by phone or fax, only between the times of 08:00 and 20:00. Clinicians will not be alerted of hyperkalemia outside of those times. Only potassium values >6.6 mmol/L are alerted to the ordering clinician 24 hours a day and 7 days a week.^43^ These guidelines are used by both Dynacare and Life Labs, which are the largest outpatient laboratory service providers in Ontario. Both companies also provide services across Canada. These lab reporting guidelines vary between provincial jurisdictions. For instance, the lab reporting guidelines by Alberta Precision Laboratories in Calgary, Alberta, requires that clinicians are notified at any time if there is a potassium value >6.2 mmol/L.^44^ Additionally, international surveys of biochemistry labs show similar discrepancies in the definition of urgent potassium values (Table 3).^45,46^ Many of the survey respondents did not provide clinical evidence for developing the selected threshold. If evidence was provided, it was based on inpatient data rather than outpatient outcomes. In fact, the international survey also highlighted how the reporting guidelines were created. There was variation including published literature, recommendations from medical staff, and international guidelines (Table 3). An international standard on reporting severe hyperkalemic values has yet to be determined.

**Table 3.** Lab Reporting Guidelines for Potassium.

Published lab reporting guidelines	Lab reporting threshold	
OAML Reporting Guidelines	>6.6 mmol/L—Level I Alert Physicians are called 24/7 as soon are result is available 6.2-6.5 mmol/L—Level II Alert Physicians are informed of the result between 8am-8pm by phone or fax	
Alberta Precision Laboratories	>6.2 mmol/L—Critical Value Physicians are called 24/7 as soon are result is available	
Published surveys by country	Lab reporting threshold	How thresholds were developed, if data available
United Kingdom	>6.2 mmol/L (range 6.0-6.5 mmol/L)	
United States of America	>6.0 mmol/L (range 5.9-6.5 mmol/L)	1/3 published literature 1/3 non-laboratory medical staff recommendations
Italy	>6.2 mmol/L (range 5.5-7.1 mmol/L)	57% published literature 37% Italian laboratory society recommendations 21% clinician opinions
Spain	>6.3 mmol/L (range 6.0-7.7 mmol/L)	52% from local data 48% published literature 10% clinician opinions
Australia	>6.2 mmol/L	62% laboratory professional experience 59% published literature 41% international guidelines 41% clinician opinions

*Note.* OAML = Ontario Association of Medical Laboratories.

Potassium reporting in special populations, such as dialysis patients, may differ. In Ontario, conventional hemodialysis occurs in hospital settings or at sites directly affiliated with a hospital.^47^ There are processes in place where a severe hyperkalemic value will be directly reported to the dialysis unit, where a change in dialysis prescription can be made. Since community laboratories are unaware which patients may be receiving home dialysis therapy, hyperkalemic values in these patients will trigger notifications to the ordering provider.

At this time, there is no evidence to support what thresholds should result in provider notification and how quickly treatment should be initiated. Developing evidence-based laboratory reporting guidelines may help balance receiving important laboratory results without causing unnecessary calls. There is clearly a discrepancy between published laboratory reporting guidelines and suggested management from expert panels. This discrepancy is highlighted in Tables 2 and 3.

## Filling the Gaps: A Call to Improve Outpatient Hyperkalemia Management

There are multiple dimensions of hyperkalemia and a standardized treatment strategy requires an interdisciplinary approach. Our group proposes that improving outpatient hyperkalemia management requires 3 strategies.

First, health care providers should only be alerted to accurate results. This necessitates a collaboration with clinical biochemists and clinicians. Currently, clinicians only receive an alert for hemolyzed samples. However, there are no standardized alerts for other causes of factitious hyperkalemia, such as delayed processing. A clinical decision-making tool that considers factors such as previous creatinine and potassium results and duration of potassium processing and analysis should be developed. These factors inform the accuracy of the reported hyperkalemia result. A population-level study to integrate these factors, amongst others, would be required to validate such a tool. This will aid in the clinical decision-making process on whether a patient needs to be urgently treated in the ED.

Second, potassium reporting guidelines should be evidence-based. The threshold for severe hyperkalemia, which must be immediately communicated to health care providers, needs to be clearly defined. This can be done with collaboration between clinical biochemists and clinicians. Currently, jurisdictions use different hyperkalemia thresholds, which may need to be altered to higher or lower values. Population level studies that focus on outpatient hyperkalemia outcomes need to be developed to determine the optimal threshold for potassium that necessitates ED treatment to avoid poor outcomes such as death. In doing so, a clinical decision alert may be incorporated on the lab report that informs providers of the likely outcome of a hyperkalemia result.

Finally, development of a standardized evidence-based guideline on how clinicians should approach outpatient hyperkalemia is in great need. Should patients with severe outpatient hyperkalemia be sent to the ED for urgent and prompt management? When is it safe to manage hyperkalemia as an outpatient with medication changes or use of potassium binders? These are common questions that health care providers face, and without any evidence-based guidance, providers must rely on their own experiences and comfort levels. A risk calculator for severe hyperkalemia can be developed that can provide clinical guidance to health care providers to refer their patient to the ED for urgent management. This calculator could be implemented as a population-level clustered randomized control trial to investigate its efficacy to reduce poor clinical outcomes.

To our knowledge, there is no systematic assessment of the consequences of different approaches to outpatient hyperkalemia treatment. Since hyperkalemia is a medical risk and psychological burden for patients, a rigorous investigation of this phenomenon is required from a health care system, provider, and patient perspective. Evidence-based guidelines on best management of outpatient hyperkalemia, from a laboratory and clinical perspective, are required to ensure optimal patient outcomes and health care utilization.
